# *PTPN11* mutation manifesting as LEOPARD syndrome associated with hypertrophic plexi and neuropathic pain

**DOI:** 10.1186/s12883-015-0310-8

**Published:** 2015-04-16

**Authors:** Marianna Spatola, Christian Wider, Thierry Kuntzer, Alexandre Croquelois

**Affiliations:** Department of Clinical Neurosciences, Lausanne University Hospital (CHUV), Rue du Bugnon 21, 1011 Lausanne, Switzerland

**Keywords:** LEOPARD syndrome, Hypertrophic nerve roots, RAS-MAPK syndromes, PTPN11 mutation, Neuropathic pain

## Abstract

**Background:**

LEOPARD syndrome (LS) belongs to the family of neuro-cardio-facio-cutaneous syndromes, which include Neurofibromatosis-1 (NF1), Noonan syndrome, Costello Syndrome, cardio-facio-cutaneous syndrome, Noonan-like syndrome with loose anagen hair and Legius syndrome. These conditions are caused by mutations in genes encoding proteins involved in the RAS-MAPK cellular pathway. Clinical heterogeneity and phenotype overlaps across those different syndromes is already recognized.

**Case presentation:**

We hereby report a heterozygous *de novo* mutation in the *PTPN11* gene (c.1403C > T) manifesting with a clinical picture of LS during childhood, and later development of neuropathic pain with hypertrophic plexi, which are typically observed in NF1 but have not been reported in LS.

**Conclusion:**

LS caused by *PTPN11* mutations may be associated with hypertrophic roots and plexi. Consequently, clinicians should be aware of the possible development of neuropathic pain and consider specific diagnostic work-up and management.

## Background

LEOPARD syndrome (LS) is defined by Lentigines, Electrocardiographic abnormalities, Ocular hyperthelorism, Pulmonary valve stenosis, Abnormal genitals, Retarded growth and Deafness, and is caused by heterozygous mutations in the protein tyrosine phosphatase, non-receptor type 11 gene (*PTPN11*). LS is a rare, autosomal dominantly inherited disease, belonging to the family of neuro-cardio-facio-cutaneous syndromes (NCFCs). These syndromes present significant genetic heterogeneity and phenotype overlaps. Here we describe a patient who arbors a heterozygous mutation in the *PTPN11* gene (c.1403C > T) manifesting with a clinical phenotype of LS associated with neuropathic pain and hypertrophic roots and plexi.

## Case presentation

**Figure 1:**
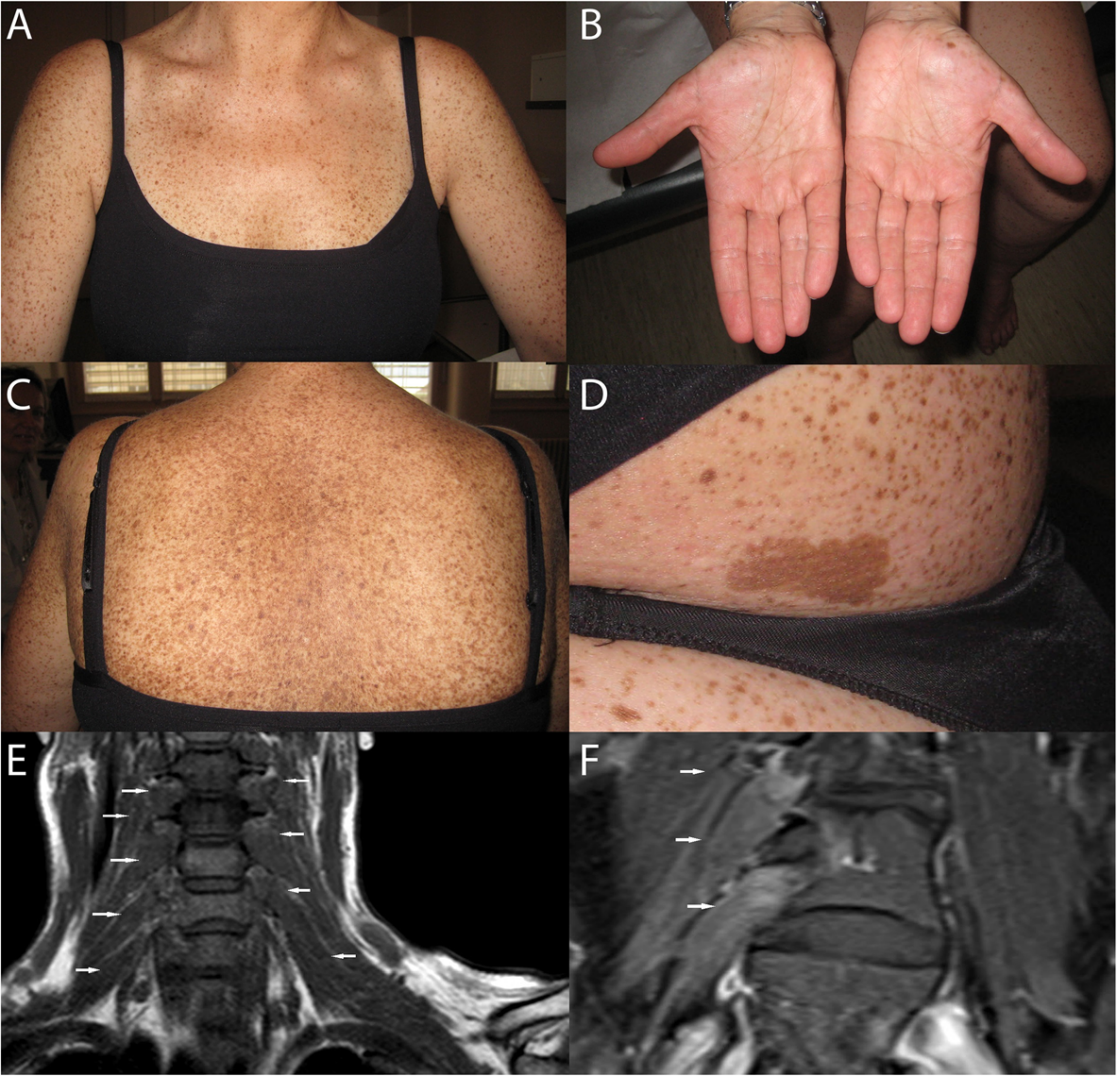
Clinical and MRI features of LEOPARD syndrome presenting with hypertrophic plexi and neuropathic pain. Patient with LEOPARD syndrome showing multiple cutaneous lentigines (**A** and **C**: trunk, **B**: palms) and café-au-lait spots, on the upper buttock **(D)**. Spinal MRI showing hyperthrophy of the cervico-brachial **(E)** and lumbo-sacral **(F)** plexi.

A Caucasian woman presented with multiple cutaneous diffuse lentigines and cafe-au-lait spots (7 spots of 0.7 to 5 cm of diameters, localized in the palms, feet, trunk and buttock) since the age of 6 years (Figure 1A-D), associated with mild bilateral ptosis, facial dysmorphism (such as prognathism necessitating surgery, macroglossia) and pulmonary valve stenosis. Her body weight and height was in the normal age-range and she had no psychomotor delay, working as a commercial manager. Ophthalmologic examination revealed multiple peripheral retinal lentigines. Family history was negative for neurological or cutaneous symptoms.

She was re-examined at the age of 41 years, related to the development of progressive painful dysaesthesias. Neurological examination revealed moderate sensory-motor deficit in all four limbs, with mild facial weakness. Conventional nerve conduction studies of the upper and lower limbs were normal, but late spinal F latencies were diffusely prolonged, suggesting diffuse proximal neuropathy. Spinal MRI revealed hypertrophic cervico-brachial and lumbo-sacral plexi and roots (Figure 1E, F). Extensive diagnostic work-up excluded other inflammatory, genetic or tumor conditions associated with hypertrophic neuropathies [1–3]. MRI of the lower limbs showed two gadolinium-enhanced T2-hyperintense nodules (not shown), suggestive of neurofibromas. Sequencing of the *NF1* gene showed no mutation (including search for intragenic rearrangements by MLPA analysis), while sequencing of the *PTPN11* gene revealed a heterozygous missense mutation in Exon 12 (c.1403C > T, p.Thr468Met), confirming the diagnosis of LS.

## Conclusion

We report a patient harboring the *PTPN11* c.1403C > T mutation which manifested with a phenotype of childhood-onset LS, fulfilling clinical criteria (multiple lentigines plus 2 major features [4]), and later development of neuropathic pain related to hypertrophic nerve roots and plexi.

The association of skin changes, facial dysmorphism and mild cardiac abnormalities are almost universally present in LS patients [4]; sensorineural hearing loss and neurological deficits (neuropsychological difficulties and rarely seizures) are present in less than a third of the cases. Most abnormalities are present at birth, however some can appear during childhood, as is often the case for lentigines, or even develop during adulthood, as for ventricular hypertrophy.

Mutations in *PTPN11* are responsible for 80% of LS cases. The c.1403C > T nucleotide substitution found in our patient is the most frequently reported mutation, and it leads to a decreased catalytic capacity of the SHP2 protein. *RAF1* and *BRAF* mutations are much less frequently encountered.

LS belongs to the NCFCs family. The most prevalent disorders of this group are Noonan syndrome and Neurofibromatosis-1 (NF1), while LS is a rarer condition. NCFCs display common clinical features such as psychomotor delay, facial dysmorphism and cardiac, cutaneous and skeletal abnormalities. Although genetic causes of NCFCs are heterogeneous, the molecular mechanisms involved alter the RAS/MAPK signaling pathway [5]. *RAS* is a human oncogene implicated in different cellular functions and its transducing signal, involving different MAP kinases, is critical for cell proliferation and survival. Thus, its disruption may result in uncontrolled cell growth and cancer. Somatic mutations in protein components of this pathway are found in various malignancies [6]. In contrast, germ-line mutations, which result in a less dysfunctional gene product, underlie the development of NCFCs.

NCFCs display significant genetic and phenotypic heterogeneity: on the one hand, mutations in the same gene may be responsible for different syndromes, as is the case for Noonan syndrome and LS, mostly caused by mutations in the *PTPN11* gene; on the other hand, dysfunction of different proteins at various levels of the RAS/MAPK cascade can lead to similar clinical presentations [7,8].

Our patient harbored a *PTPN11 de novo* mutation manifesting as LS associated with nerve and roots hypertrophy and neuropathic pain. Although these latter changes, associated with gadolinium-enhanced deep nodules evoking neurofibromas, are typical findings of NF1, our subject presented no *NF1* gene mutations. Recently, three cases of *PTPN11* mutations were reported with early cutaneous, but no neurological, features of NF1 [9]. Our patient showed cafe-au-lait spots, however without other dermatological hallmarks of NF1, such as skinfold freckling or cutaneous neurofibromas. Some authors suggested that NF1 can be misdiagnosed if based solely on the presence of these skin abnormalities, which can be encountered in other conditions and disappear with growth [10].

Our observation underscores that LS caused by *PTPN11* mutations could be associated with a proximal radiculoplexopathy. This finding is of clinical relevance: while neurologic complications are common in NF1, they are not routinely screened for in LS patients. Thus, clinicians should question adult LS individuals about the development of neuropathic pain and neurological deficits, and perform careful physical examination and consider spinal MRI in search of plexi/root hypertrophy and neurofibromas.

Neurogenic pain can significantly impact quality of life, and its management may be challenging. In order to address different important aspects related to pain, especially in the context of NCFCs, a multidisciplinary approach is recommended, associating physical therapy with medical (including analgesics, opiates, antiepileptic drugs, topic anesthetics, antidepressants, or a combination) and psychological treatments [11]. For example, a multimodal approach including a relaxation response resiliency program (3RP) has been recently used in a pilot study involving patients with NF1, NF2, and schwannomatosis, with encouraging results in terms of coping strategies and quality of life [12]. The use of oral ketamine or bevacizumab has also been suggested as a therapeutic option in refractory cases of NF1 or NF2, respectively, but its efficacy only relies on single case reports [13,14]. By contrast, surgery should be considered in patients with nerve or spine compression by plexiform neurofibromas.

Additionally, oncologic screening should be considered in LS patients presenting deep nodules suggestive of neurofibromas, particularly in light of the risk for malignant transformation, in analogy to what is observed in NF1 patients [15]. This is particularly important in the perspective of promising target-based therapies acting directly on the RAS/MAPK signaling pathway, such as MEK and RAF inhibitors [16]. Although it has been suggested that these molecules may play a role in controlling tumor progression in various conditions associated with neurofibromatosis [16], their effectiveness in neurogenic pain control seems to date less prominent [17–19].

## Consent

Oral informed consent was obtained from the patient for publication of this case report and any accompanying images. Since the patient was not reachable, a written consent could not be obtained.

## Abbreviations

LS: LEOPARD syndrome
PTPN11: Tyrosine-protein phosphatase non-receptor type 11
NCFCs: Neuro-cardio-facio-cutaneous syndromes
NF1: Neurofibromatosis type 1
NF2: Neurofibromatosis type 2
